# Oxidative Stress and Neurodegeneration: Insights and Therapeutic Strategies for Parkinson’s Disease

**DOI:** 10.3390/neurolint16030037

**Published:** 2024-04-29

**Authors:** Erjola Bej, Patrizia Cesare, Anna Rita Volpe, Michele d’Angelo, Vanessa Castelli

**Affiliations:** Department of Life, Health and Environmental Sciences, University of L’Aquila, 67100 L’Aquila, Italy; erjola.bej@graduate.univaq.it (E.B.); patrizia.cesare@univaq.it (P.C.); annarita.volpe@univaq.it (A.R.V.); michele.dangelo@univaq.it (M.d.)

**Keywords:** neurodegeneration, dopamine, mitochondria, neuroinflammation, neuroprotection, alpha-synuclein, reactive oxygen species, phytotherapy

## Abstract

Parkinson’s disease (PD) is a progressive neurodegenerative condition marked by the gradual deterioration of dopaminergic neurons in the *substantia nigra*. Oxidative stress has been identified as a key player in the development of PD in recent studies. In the first part, we discuss the sources of oxidative stress in PD, including mitochondrial dysfunction, dopamine metabolism, and neuroinflammation. This paper delves into the possibility of mitigating oxidative stress as a potential treatment approach for PD. In addition, we examine the hurdles and potential of antioxidant therapy, including the challenge of delivering antioxidants to the brain and the requirement for biomarkers to track oxidative stress in PD patients. However, even if antioxidant therapy holds promise, further investigation is needed to determine its efficacy and safety in PD treatment.

## 1. Introduction

Neurodegenerative disorders are a major clinical distress, especially for elderly people [1]. Parkinson’s disease (PD), one of the main neurological disorders associated with the Lewy bodies and loss of dopaminergic neurons in the *substantia nigra pars compacta* (SNpc), continues to pose significant challenges in the field of neurology [2,3]. The loss of SNpc dopaminergic neurons leads to a dopamine deficit, which is the main cause of motor symptoms in PD patients [4]. Clinical manifestations of this disease involve resting tremor, postural instability, rigidity, and slowness or absence of voluntary movement, but they can also be accompanied by cognitive and behavioral problems [5]. Unfortunately, PD is not recognized in its early stage in most cases [6]; the visible symptoms do not develop until we have lost almost 80% of dopaminergic neurons [7]. Accumulating evidence seems to connect oxidative stress with dopaminergic neurodegeneration [8,9]. Dysregulation of cellular redox activity leads to an outweigh production of reactive oxygen species (ROS), much more than the endogenous antioxidant can clear out. ROS accumulation is the first step, followed by neuronal damage [10], and it may cause oxidative damage to DNA, protein, or lipids [11]. Several data collected from PD patients in their early stage reveal that oxidative stress is present from the beginning of the disease, which leads us to think that ROS may be the cause of dopaminergic neuronal damage and loss other than being the response to neurodegeneration [12]. Despite advances in understanding PD pathophysiology, effective treatments that can halt or reverse the progression of PD remain elusive. Recent research has increasingly pointed to oxidative stress as a key contributor to the development and progression of PD. This review aims to explore the potential of targeting oxidative stress as a therapeutic strategy for the prevention and treatment of PD.

## 2. Pathophysiology of PD 

The pathophysiology of PD is linked to the degradation of dopaminergic neurons in the brain, which results in motor cortical excitation and inhibition. Underlying the motor symptoms is a loss of dopamine input into the basal ganglia, caused by the progressive death of dopamine neurons within the *substantia nigra*. Pathological hallmarks of PD include neuroinflammation, the degeneration of dopaminergic neurons in the *substantia nigra pars compacta*, and the accumulation of misfolded α-synuclein proteins as intra-cytoplasmic Lewy bodies and neurites [13]. Dysregulation of glial cells results in the disruption of homeostasis, leading to a chronic pro-inflammatory, deleterious environment. Recent studies reported a role for peripheral immune cells, in particular T lymphocytes, in the pathogenesis of PD [14]. The majority of PD cases are idiopathic or sporadic, with no known cause. However, there is increasing evidence for the role of both genetic and environmental influences.

### 2.1. Clinical Features of PD

PD is characterized by motor and non-motor symptoms. From the motor point of view, parkinsonism is based on four main features: bradykinesia, rest tremor, rigidity or inflexibility, and postural and walking abnormality. 

Bradykinesia refers to progressively slower movements [15]. It is very important for the treatment to tell the difference between true bradykinesia and the simple slowness of patients suffering from decreased muscle power or reduced motivation in cases of patients suffering from depression. Other clinical displays are hypomimia, which is reduced face expression and eye blinking, hypophonia, which consists of a reduction in the volume of the voice, micrographia, which is progressively smaller handwriting, and difficulty swallowing [16]. 

On the other hand, the rest tremor is a repeated and periodic unconscious movement that is observed when the affected body part, the hand for example, rests on the surface because of not having to use gravitational force [17]. It vanishes with the active movement to reappear after a few seconds if the hand is stretched out. 

Rigidity refers to increased muscle tone, involving both flexor and extensor muscle groups [18]. This rigidity does not increase with higher mobilization speed. 

Postural and walking abnormality are adaptations where PD patients have to adopt a stopped posture due to the loss of postural reflexes [19]. Parkinsonian gait is slow, and it is identified by short steps and decreased arm swing, while the freezing of gate can occur, most of the time, in narrow or overcrowded places [20,21]. 

**Figure 1:**
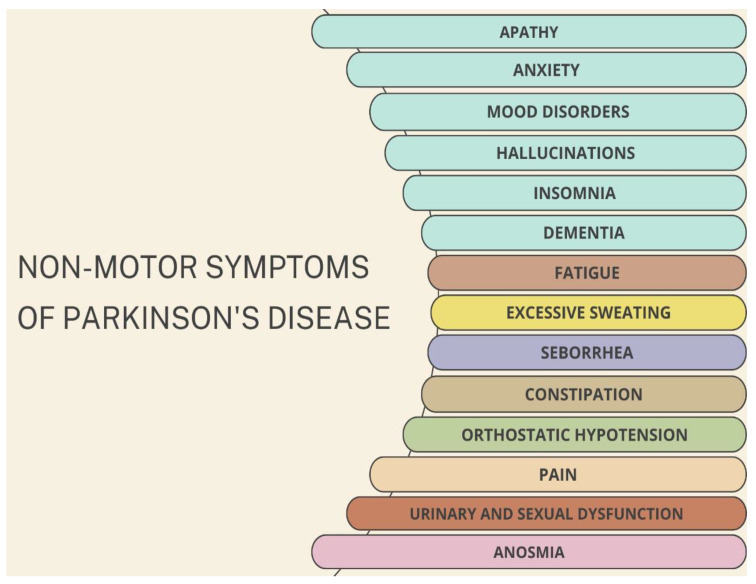
Non-motor symptoms of PD include neurological effects such as apathy, anxiety, mood disorders, hallucinations, dementia, insomnia, and pain. Other symptoms are related to olfactory system such as anosmia and gastrointestinal effects, such as constipation. Some affected people also experience orthostatic hypotension, urinary and sexual dysfunctions, and sustained activity of sweat and sebaceus glands.

Even though PD has always been recognized as a motor disorder, in recent years, there has been an increase in interest in non-motor symptoms (such as anosmia, mood disorders, hypotension, fatigue and excessive sweating, urinary and sexual dysfunction), not only because their recognition is helpful for diagnostic purposes but also because treating these symptoms can improve quality of life [22,23,24] (Figure 1).

Some of these non-motor symptoms may be present sometimes for years or decades before any of the classical motor signs manifest. In the meantime, other features like dementia or hallucinations occur later on with the progression of PD, but this might also be useful to distinguish PD from other disorders [25,26].

The underlying mechanisms of PD onset and progression are not really clear, but neuroinflammation, neuronal cell rearrangement, and oxidative stress are strongly involved [27]. In this review, we focus on analyzing the potential mechanisms of interrelation between oxidative stress and PD, highlighting the most promising compounds targeting it.

### 2.2. Mitochondria and Oxidative Stress

Some of the most important determinant factors in dopaminergic neuronal susceptibility in both juvenile onset PD and idiopathic forms of the disease are mitochondrial dysfunction, neuroinflammation, and also environmental factors [27]. In both cases, oxidative stress is thought to be the principal instrument associated with cellular dysfunction and then cell death. Reactive oxygen species (ROS) production has been reported in vivo in all body cells and tissues, but oxidative stress happens when there is a disbalance between ROS production and cellular antioxidant activity, crucial in regulating the redox state of the body [8]. Oxidants and superoxide radicals are produced in the mitochondria, which is the main site of ROS generation inside the cell; on the other hand, ROS reduction may occur in different cellular compartments through different antioxidants enzymes [28].

**Figure 2:**
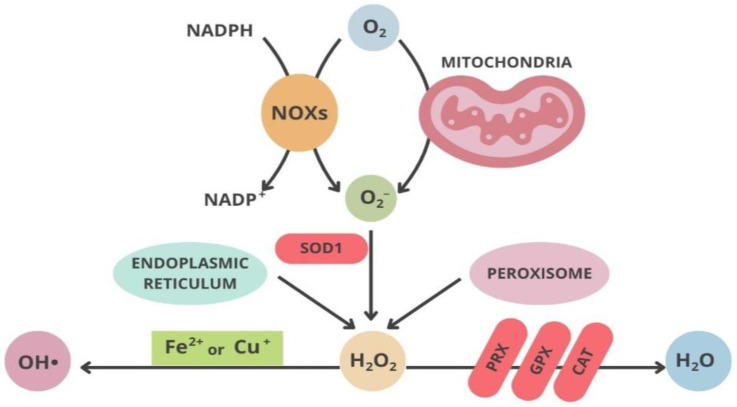
Schematic representation of the main pathways in oxidative stress.

In physiological conditions, mitochondria are a key intracellular origin of ROS during aging processes [29], while, in pathological conditions, we observe a higher production of ROS in PD due to mitochondrial dysfunctions [30]. The necessary energy for neural activity is provided by oxidative phosphorylation, a process that is the starting point of superoxide and hydrogen peroxide, resulting in the development and the progression of the disease [31]. According to Henry J. H. Fenton, hydrogen peroxide may trigger the formation of hydroxyl radicals OH· [32]. Mitochondria contain superoxide dismutase (SOD1) enzyme, while in the meantime, we find SOD2 mostly in the cytosol; both enzymes can transform the $O_{2}^{-}\cdot$ into a less dangerous hydrogen peroxide ($H_{2}O_{2}$) [33]. $H_{2}O_{2}$ obtained by SOD1 and SOD2 is again transformed into harmless $O_{2}$ and $H_{2}O$ [33] (Figure 2). Since $H_{2}O_{2}$ has a considerable rate of spreading and distribution from mitochondria to other cell compartments, it can play a role as a redox-directing molecule [34,35]. After oxidative modifications, proteins may alter their physiological functions [36]. 

One of the main causes of ROS hyper production in PD patients is deficiencies in complex I of the respiratory chain in the mitochondria [37]. Therefore, all the compounds that are complex I inhibitors, such as 1-methyl-4—phenyl-1,2,3,6-tetrahydropyridine (MPTP) or rotenone, show neurotoxicity in regard to DA neurons because they might cause the overproduction of ROS [38]. MPTP crosses the BBB, is taken up by astrocytes, and is oxidized to 1-methyl-4-phenylpyridinium (MPP+) by monoamine oxidase-B (MAO-B). It then accumulates in the mitochondria to inhibit complex I in the METC (mitochondrial electronic transport chain), which results in not only decreased ATP production but also increased ROS generation [8]. Rotenone, on the other hand, is another mitochondrial complex I inhibitor that causes oxidative damage to proteins and Lewy body-like inclusions; it also inhibits the METC, leading to ATP depletion [39,40]. Since demand surpasses supply in METC dysfunction, we have a premature increase in electron transfer to $O_{2}$ generating $O_{2}^{-}$, which is the cause of oxidative stress to the DNA, proteins, and lipids [41]. Other studies show that mutations of any kind in genes like parkin, DJ-1, and PTEN-induced kinase 1 (PINK1) are tightly associated with oxidative stress and dopaminergic cell damage and mitochondrial dysfunction [42,43]. Parkin and PINK1 are localized in the mitochondria, and their function is to repair the dysfunctional mitochondria, improving its health; they might also be able to mediate the mitochondria [44,45]. When there are parkin mutations, mitochondrial complex I activity is weakened and damaged [46]. By protecting the mitochondria, the overexpression of Parkin can contrast the DA neuronal cell loss caused by the MPTP [47]. A lack of or deficient parkin can result in mitochondrial malfunction, as well as in Drosophila, according to Saini’s study [48]. PINK1 may have neuroprotective effects on dopaminergic cells because it is believed to act as a mitochondrial Ser/Thr protein kinase, playing a crucial role in sustaining the mitochondrial membrane potential and morphology [49]. Deficient PINK1 or a lack of it can lead to mitochondrial defects, respiratory chain deviations, reduction in complex I activity, enhanced vulnerability to oxidative stress, and loss of SNpc DA neurons [50,51]. Amplifying the expression of PINK1 in terms of quality or quantity can resolve most of these deficiencies [52].

The agglomeration of the protein α-synuclein as an intracellular aggregate is a characteristic attributed to PD and associated with the reduction in mitochondrial complex I activity, leading to upraised ROS generation and neuronal death [53]. This mitochondrial dysfunction, in mice overexpressing α-synuclein, is the first process that takes place, then several months after, the appearance of striatal DA loss is observed [54]. Oxidative stress contributes to the intake, agglomeration, and oligomerization of extracellular α-synuclein in oligodendrocytes. Oxidative stress upgrades each consecutive process of intaking, agglomeration, and oligomerization of α-synuclein within the oligodendrocytes, and this is a characteristic of neurodegenerative disorders [55]. Post-translational changes in the α-synuclein, which are caused by high levels of oxidative stress, lead to DA loss [56].

DJ-1 is a mitochondrial protein that in physiological conditions tends to bind to the subunits of the mitochondrial complex I, regulating its activity [57]. A slight part of the DJ-1 is also found in the mitochondria matrix [58]. The mitochondrial localization of DJ-1 leads to intensified neuroprotection [59].

Several factors could contribute to the mitochondrial dysfunction associated with PD. These include the loss of mitochondrial biogenesis, overproduction of ROS, dysfunction in mitophagy, compromised trafficking, deficits in the function of the ETC, changes in mitochondrial dynamics, imbalances in calcium, and others [60]. In PD, a key factor is the malfunctioning of mitochondria, especially in the electron transport chain (ETC) [60,61]. Indeed, this disease is often associated with problems in complex I of the ETC, which is vital for producing energy in mitochondria, a process crucial for the health of neurons. Complex I deficiencies of the respiratory chain account for the majority of unfavorable neuronal degeneration in PD.

As mentioned, there are over 20 genes linked to PD that have been identified in recent decades. Some of these genes, such as PRKN, PINK1, and DJ-1, directly affect mitochondrial functions. Other genes associated with PD, like LRRK2, SNCA, and GBA1, regulate functions related to lysosomes, lipid metabolism, or protein aggregation, functions which have been shown to indirectly impact the ETC.

Recent discoveries of CHCHD2 and UQCRC1, which are essential for the functioning of complex IV and complex III, respectively, provide solid evidence that PD is more than just a complex I disorder [61]. These findings suggest a complex relationship between the malfunctioning of mitochondria, particularly in the ETC, and the development of PD. However, the exact mechanisms are still not fully understood, and more research is needed in this area.

Another contributor of oxidative stress observed in PD is iron accumulation in SNpc. Specifically, PD patients have elevated levels of brain iron, especially in the nigrostriatal dopaminergic system, which is likely caused by abnormalities in various iron metabolism-related proteins, leading to disruptions in iron distribution, transport, storage, and circulation [62,63]. Excessive iron can induce oxidative stress and iron-related cell death, thus exacerbating mitochondrial dysfunction and contributing to the progression of PD pathology [62]. In cells, free ferrous irons (Fe^2+^) react with hydrogen peroxide (Fenton reaction), producing harmful ferric irons (Fe^3+^) and ROS, which damage cellular components [63].

Abnormal iron deposition can also lead to the death of dopaminergic neurons in the SNpc through various pathways, such as by inducing mitochondrial dysfunction and promoting iron-dependent cell death [64,65]. Interestingly, Magnetic Resonance Imaging (MRI) studies have indicated that the characteristics of iron deposition in the brains of PD patients vary. Iron deposition correlates with the clinical symptoms of PD, and patients with different disease courses and clinical presentations display distinct patterns of iron deposition. These findings suggest a complex relationship between iron deposition/ferroptosis in the SNpc, oxidative stress, and the pathogenesis of PD. However, the exact mechanisms are still not fully understood, and further research is necessary.

ROS accumulation can promote cell death in different ways, including apoptosis, autophagic, and cytoplasmic cell death [66,67]. Pro-apoptotic proteins, such those of the family of Bcl-2 proteins (Bax, Bak, Bad, Bim), undergo conformational changes after being set in motion after ROS accumulation. These pro-apoptotic proteins need to be relocated into the mitochondrial membranes so that they can obtain the liberation of apoptogenic factors such as cytochrome c [68].

Disruptions in dopamine metabolism can lead to an increase in ROS, contributing to oxidative stress. This oxidative stress can cause damage to various cellular components, including lipids, proteins, and DNA, ultimately leading to cell death. This is particularly detrimental in the SNPc region, where dopamine-producing neurons are located [69]. Moreover, certain proteins, such as α-synuclein, have been found to interact with both dopamine metabolism and oxidative stress pathways. In particular, the aggregation of α-synuclein, a characteristic feature of PD, is associated with increased oxidative stress and disruptions in dopamine metabolism. 

A recent study by Li and collaborators highlighted the interconnection of different neuronal events, emphasizing the importance of considering both alterations in DNA/RNA oxidative damage and vesicular monoamine transporter 2 (VMAT2) densities in the caudate and putamen from patients with PD [69]. This study, for the first time, investigated the interrelationship of dopamine and oxidative damage in the striatum of neurodegenerative brains [69]. 

Understanding the interplay between dopamine metabolism and oxidative stress in PD could potentially open up new avenues for therapeutic interventions. For instance, antioxidants that can reduce oxidative stress or drugs that can modulate dopamine metabolism might be beneficial in treating PD.

### 2.3. Neuroinflammation in PD

Neuroinflammation is also a feature of both familial and sporadic forms of PD. ROS may affect mitochondrial functionality, leading to alterations in redox homeostasis, dysfunctional mitochondria, and neuroinflammation. Neuronal loss in PD patients is mostly caused by neuroinflammation controlled especially by the microglia, being the cells that repair damage, and in a lower grade by astrocytes and oligodendrocytes microglia mostly, but astrocytes and oligodendrocytes also control the chronic neuroinflammation which accompanies neuronal loss in PD patients [70]. Microglia polarization and pro-inflammation has been found to occur at a higher density in the SNpc of PD animal models [71]. Exposure to certain environmental toxins can cause microglia to enter a hyperactivated state and release ROS [72]. ROS production and release can also cause the activation of enzymes such as NADPH oxidase (NOX2) that damage the nearby neurons [73].

Mechanisms related to immune response can act as modifiers at different steps of the neurodegenerative process. The aberrant activation of glial cells and other components of the immune system create a vicious circle in which neurodegeneration and neuroinflammation nourish each other. Specifically, oxidative stress and neuroinflammation are interrelated and contribute to the progression of PD [74]. They create a self-sustaining loop where neurodegeneration and neuroinflammation feed off each other, exacerbating the progression of the disease [75]. In particular, defective mitochondria can trigger an inflammatory response, a process that involves the release of mitochondrial contents into the cytoplasm or the extracellular environment. This release can occur when the mitochondria’s outer and inner membranes are damaged under certain stress conditions. The released mitochondrial components are identified by pattern recognition receptors (PRRs) as DAMPs, signaling cellular damage and initiating the innate immune response [76,77,78]. The glycolytic enzyme hexokinase (HK), located on the outer mitochondrial membrane, has been identified as the PRR for n-acetylglucosamine. Upon the detection of released n-acetylglucosamine, HK detaches from the mitochondrial outer membrane and releases mtDNA, which activates the NLRP3 inflammasome [79].

Research has shown that human THP1 macrophages treated with the mitochondrial complex 1 inhibitor rotenone exhibit a dose-dependent increase in IL-1β secretion. This is accompanied by a loss of mitochondrial membrane potential and ROS production. However, when NLRP3 was removed from THP1 cells, respiratory chain inhibitors did not induce IL-1β or caspase-1 secretion. This suggests that mitochondrial dysfunction stimulates (chronic) inflammation through NLRP3 inflammasome-dependent inflammatory pathways [80].

It has also been reported that mitochondrial ROS, mtDNA, and particularly oxidized mtDNA trigger the activation of NLRP3 inflammasomes [81]. The NLRP3 inflammasome complex serves as a sensor of mitochondrial dysfunction, and its activation leads to the production of IL-1β [74]. Inflammatory cytokines can induce mitochondrial impairment and ROS production, creating a self-toxic feedback loop. Furthermore, the systemic injection of LPS leads to the region-specific expression of neuroinflammatory markers and alters mitochondrial activity and oxidative phosphorylation in normal mouse brain regions [82]. Given the strong correlation between ATP levels and neuroinflammatory markers, it is believed that the underlying mechanism of the acute neuroinflammatory response following systemic LPS injection may involve differential changes in oxidative stress, mitochondrial activity, and oxidative phosphorylation [82].

MPTP (1-methyl-4-phenyl-1,2,3,6-tetrahydropyridine) is a complex I inhibitor in mitochondria, which increases the levels of inflammatory cytokines and leads to microglial activation in mice [83] and monkeys [84]. According to Joglar’s study, we have been led to believe that the inflammatory response in the MPTP model might be mediated by the brain peptide, angiotensin, which is considered to be very important regarding the inflammation and oxidative stress activators [85]. An important role in rotenone-induced neuronal degeneration through NADP is played by the microglia and by the inhibition of mitochondrial complex I of the nerve cells [86,87].

Neuromelanin, which is produced by the oxidation of dopamine, is responsible for SNpc dark pigmentation and appearance [88]. Having high levels of catecholamine metabolism is correlated with high levels of neuromelanin that is responsible for inducing neuroinflammation in PD because the release of neuromelanin from the dying dopaminergic neurons can increase the vulnerability of the other in-good-health dopaminergic neurons to oxidative-stress-mediated neuron’s death [89,90]. Furthermore, increased levels of iron and the dysfunction of iron homeostasis can result in increased ROS production. Iron homeostasis itself is modulated by the angiotensin; disbalances can intensify the microglial inflammatory response and the advancement of dopaminergic degradation [91].

## 3. Treatments Targeting Oxidative Stress in PD

The most frequent therapy for PD is the one based on medications that treat the lack of dopamine. Levodopa is the precursor of dopamine [6]; it is converted into dopamine by the enzyme dopa-decarboxylase. Before reaching the CNS, Levodopa can be decarboxylated. Therefore, when using it along with carbidopa, which is an inhibitor of the enzyme L-amino-acid decarboxylase, but in the meantime does not pass the BBB, the unchanged levodopa can penetrate in the CNS and be the precursor of dopamine [92]. Levodopa influences cholinergic, GABAergic, and glutamatergic neurons, while abnormal serotoninergic transmission is involved in side effects of levodopa like dyskinesia and psychosis [93]. Levodopa is one of the most important drugs used in controlling the symptoms of PD [94]. After 5 years of treatment, most patients experience side effects such as motor oscillations and dyskinesias, which are dose-dependent and associated with the duration of treatment [95,96]. There are different types of dyskinesias induced by levodopa, such as ‘peak-dose dyskinesias’, ‘biphasic dyskinesias’, and ‘wearing off’ dyskinesias [97]. Therefore, therapeutical strategies are designed to delay the onset of the collateral effects of the levodopa [98]. Young patients receiving a PD diagnosis are apparently more likely to emerge levodopa-induced dyskinesias [99,100]. Amantadine and several other drugs may be used in improving levodopa-induced dyskinesia without having to reduce the levodopa dosage [97]. Furthermore, we could use a COMT inhibitor in addition to prevent the degradation of levodopa and to allow for the crossing of a higher concentration of it through the BBB, or a dopamine agonist inhibitor which can activate the dopamine receptors, in order to manage the motor complications and issues of the levodopa [101].

In the context of PD, the presence of enzymes that produce ROS, such as monoamine oxidases (MAOs), makes dopaminergic neurons particularly vulnerable to oxidative stress. Indeed, MAO (monoamine oxidase) isoforms, including MAOA and MAOB, play a crucial role in the development and progression of PD; MAOs are enzymes that catalyze the oxidative deamination of amines such as dopamine, norepinephrine, and serotonin [6]. The oxidative metabolism of dopamine by MAOs leads to ROS generation and cell death [102]. Therefore, inhibitors of MAO-B, selegiline, and rasagiline are also available as PD medications because they can protect neurons against the oxidative damage induced by the metabolism of dopamine [6].

In the study conducted by Odunze, mice deficient in vitamin E were vulnerable to MPTP toxicity [103]. The results of other studies showed that vitamin E can interfere with iron accumulation, which could be the cause of neuronal damage or neuronal loss in the case of PD patients [104] (Table 1). Therefore, administering vitamin E might be a prospective target in slowing the progressive loss of the neuronal cells and advancements in PD [105] (Table 1). Not only vitamin E but also creatine play an important function in suppressing iron accumulation [105]. Creatine and cyclocreatine, administered orally, can rehabilitate the MPTP-induced loss of dopamine in mice, and thereby can be applied as neuroprotective agents [106] (Table 1). In a clinical study of 200 patients suffering from PD and being diagnosed within the last 5 years, the daily administration of creatine resulted in ameliorated social difficulties [105].

Another promising agent in PD is represented by coenzyme Q10 (CoQ10) and its derivative. CoQ10 is a potent antioxidant that if taken as a priming treatment can inhibit ROS generation, sustain the mitochondrial membrane potential and mechanism, and, therefore, be used as a possible antioxidant therapy in neurodegenerative diseases [107] (Table 1). CoQ10 was able to decrease apoptosis in a mouse model of PD (rotenone-induced) [108]. CoQ10 protected against MPTP-induced loss of dopamine and MPTP toxicity in aged mice [109]. A multicenter, randomized, dosage-ranging trial, conducted in untreated patients with early PD, showed that the higher the dose of CoQ10 taken, the greater the benefits of slowing down the decline of PD [110] (Table 1). Interestingly, CoQ10 in association with creatine showed a better influence on regenerating and protecting the dopaminergic neurons compared to the administration of CoQ10 alone [111]. Another CoQ10 analogue, Idebenone, has passed phase III and new applications in clinical trials for PD [112].

MitoQ, also known as mitoquinonemesylate, is a powerful mitochondrial antioxidant also studied for its potential in treating PD. MitoQ showed promise in preclinical studies of PD. Indeed, in in vitro models of PD (6-hydroxydopamine and MTPT-induced PD), MitoQ exerted protective activity, reducing mitochondrial fragmentation and diminishing the activation and translocation of Bax [113,114] (Table 1).

In PD in vivo models, the oral administration of MitoQ increased the MPTP-induced depletion of dopamine, the dopamine metabolite DOPAC, and homovanillic acid in striatum comparable to control-treated levels; restored tyrosine hydroxylase expression in *striatum* and *substantia nigra* neurons of PD-treated mice; ameliorated behavioral performances; and diminished the unpaired electron spin resonance spectra [114].

On the other hand, in clinical studies, MitoQ was not so promising. Specifically, a double-blind, placebo-controlled study that enrolled 128 newly diagnosed untreated patients with PD was conducted. The study explored the hypothesis that, over 12 months, MitoQ would slow down the progression of PD, as measured by clinical scores, particularly the Unified Parkinson Disease Rating Scale. However, the study showed no difference between MitoQ and placebo on any measure of PD progression [115].

More extensive studies, including other clinical trials (using, for example, different concentrations and time points), are necessary to fully understand the potential benefits and risks of MitoQ in the treatment of PD.

There are several phytochemicals which have been studied for their potential therapeutic benefits in PD. These substances may have neuroprotective mechanisms targeting mainly oxidative stress and mitochondrial impairment but also neuroinflammation, apoptosis, abnormal protein accumulation, and neurotrophic support [116,117,118,119]. Among flavonols, quercetin showed a strong protective effect in a 6-OHDA-induced PD model, boosting mitochondrial activity, counteracting oxidative stress, and decreasing synuclein accumulation [120] (Table 1). Interestingly, the oral administration of this dietary flavonol ameliorated motor and cognitive performances in PD rat models, reducing oxidative stress, synuclein aggregation, and neuronal death and improving neurotrophic support and neuronal firing frequency [121,122,123,124].

**Table 1:** Antioxidants that target oxidative stress. (a) Vitamin E [104,105], (b) Creatine [105,106], (c) CoQ10 [107,110], (d) MitoQ [113,114], (e) Quercetin [120].

Antioxidants	Targeting Oxidative Stress	Reference
(a) Vitamin E 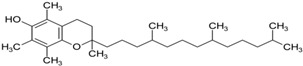	Interference with iron accumulation reducing neuronal damage and slowing down the progression of PD; protection against iron and MPTP-induced neurodegeneration in mice.	Lan and Jiang, 1997 Jin et al., 2014
(b) Creatine 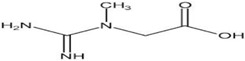	Neuroprotective agents resulting in ameliorated social difficulties; protection against MPTP-induced dopamine reduction in mice.	Jin et al., 2014 Matthews et al., 1999
(c) CoQ10 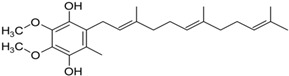	Inhibition of ROS generation, sustain of mitochondrial membrane potential, slowness of the decline of PD. It slows the decline in subjects with early PD.	Somayajulu et al., 2005 Shults, 2002
(d) MitoQ 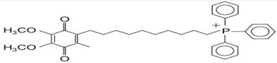	Reduction in mitochondrial fragmentation, diminishing the activation and translocation of Bax., inhibition of MPTP-induced neurotoxicity in mouse models.	Solesio et al., 2013 Ghosh et al., 2010
(e) Quercetin 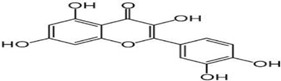	Reduction in synuclein aggregation, boost of mitochondrial activity, depletion of oxidative stress	Bayazid and Lim, 2022

The cellular redox equilibrium depends also on the use of active exogenously antioxidants [125]. Nanocarriers have been created with the main focus on transporting antioxidant molecules, improving properties such as solubility, bioavailability, clearance, half-life, stability, and bioactivity [126]. In fact, liposomes containing SOD appear to be in a higher grade of neuroprotection since the nanoparticles contribute to the improvement in stability and have risen the cellular uptake of the enzyme [127,128]. Lipid-based nanocapsules can protect the compound from enzymatic degradation, leading to higher bioavailability, targeted delivery, and reduced toxicity; therefore, they were tested by Huang et al. by encapsulating catechin, resulting in better brain distribution [129]. On the other hand, polymer-based nanoparticles encapsulating curcumin in PLGA by Tsai and collaborators were used, resulting in 22-times higher bioavailability compared to the administration of the free one and not only this, but also higher absorption, causing higher antioxidant activity [130]. From the family of catechins, epigallocatehin-3-gallate (EGCG) can be encapsulated in these polymeric nanoparticles, and according to Zhang et al.’s research, the release rate counts for 24 h in comparison the 4 h of the non-encapsulated form [131].

Another approach might be to combine SOD and catalase genes with viral vectors to form these nanocomplexes that multiplicate the enzymatic gene expression [132]. Some of the polymeric carriers such as polyethylene glycol (PEG) have some intrinsically antioxidants properties; not only so, but they can also amplify the antioxidant properties of the enclosed agent itself [133]. The carboxyl groups of citric acid, for example, can function as chelators of metal ion and, as a consequence, can detoxify one or more of the ROS subspecies [134]. SOD can also be successfully transferred using inorganic carriers, including mesoporous silica nanoparticles (MSNs), which present a high surface area, are stable under biological conditions, have low toxicity, and higher specificity [135].

On the other hand, nanogels can be additionally improved so that various antioxidants can be delivered to the brain to treat neurodegenerative diseases [136]. Nanogels of hyaluronic acid, for example, have been used to transfer curcumin and EGCG [137]. Chitosan is another polymer that can be used as a nanocarrier, but its behavior acting against free radicals depends on its molecular weight and concentration [138].

Exosomes, some extracellular vesicles, can penetrate the BBB, and, therefore, they can transport different neuroprotective agents in the brain [139,140]. Since they are cell-derived, the exosomes are immune-compatible and present reduced clearance and degradation [124]. Another way to pass through the BBB is by using viral vectors [141]. Mammalian viruses have a higher risk of immunogenicity compared to viruses isolated from plants of bacteria which have less chance of provoking side effects [142]. All these approaches aim to ameliorate the transporting of antioxidant compounds to reduce oxidative stress and mitochondrial impairment, thus slowing down PD progression. However, more research is necessary to fully understand their effectiveness and potential secondary effects.

## 4. Discussion and Conclusions

PD pathology is complex because it includes a combination of genetics, epigenetics, and environmental factors that may influence the development of the disease and its progression. Many non-motor symptoms, such as anxiety, pain, fatigue, orthostatic hypotension, and apathy, play an important role in diagnostic purposes because they might be present many years before their actual motor signs are revealed. Various data collected from PD patients in their early onset reveal that oxidative stress is present from the very beginning of the disease, which leads us to think that ROS may be the cause of dopaminergic neuronal damage and loss, other than being the response to neurodegeneration. Important contributing factors are mitochondria dysfunction, tau protein, and its abnormal hyperphosphorylation; tau protein is associated with alpha-synuclein and neuroinflammation. It might be challenging to decide whether oxidative stress is the precursor of these dysfunctions, or whether it is a consequence. Despite an intensive investigation into the PD mechanism, unfortunately, the disease is still not curable. The oxidative stress pathway has given rise to numerous novel pharmacological therapies that may provide a new avenue for neurodegenerative disorders, including PD. For example, CoQ10, SKQ (plastoquinone), MitoQ, vitamin E, and bioactive molecules have indeed been examined. Moreover, natural phytochemicals are being explored as novel therapeutic strategies to prevent and treat PD. These compounds often have antioxidant properties, which can help mitigate the oxidative stress associated with PD. It is important to note that while these strategies show promise, more research is needed to fully understand their efficacy and safety in the treatment and prevention of PD. Always consult with a healthcare provider before starting any new treatment regimen. Future studies should take into consideration research on the development of new methods to identify the disease from the pre-symptomatic stages and the development of new drugs which have neuroprotective effects, that can not only reduce disease progression but also have the minimum number of side effects.

## 5. Material and Methods

The literature reviewed included preclinical and clinical studies. We searched the literature in Google scholar, PubMed, and PMC free articles published up until February 2024. The research queries included the following terms: Parkinson’s disease, mitochondria dysfunction, alpha-synuclein, Tau protein, and abnormal hyperphosphorylation. More than 223 studies were identified, of which 142 were suitable for this article review. The figures and tables were all made from graphic software and are original.

## Data Availability

No new data were created or analyzed in this study. Data sharing is not applicable to this article.
